# Black-White differences in the effect of baseline depressive symptoms on deaths due to renal diseases: 25 year follow up of a nationally representative community sample

**DOI:** 10.12861/jrip.2015.27

**Published:** 2015-12-05

**Authors:** Shervin Assari, Sarah Burgard

**Affiliations:** ^1^Department of Psychiatry, University of Michigan, Ann Arbor, Michigan, USA; ^2^Center for Research on Ethnicity, Culture and Health, School of Public Health, Ann Arbor, University of Michigan, USA; ^3^Department of Sociology, University of Michigan, Ann Arbor, Michigan, USA; ^4^Department of Epidemiology, School of Public Health, University of Michigan, Ann Arbor, Michigan, USA

**Keywords:** Ethnic groups, Population groups, African Americans, Depression, Mortality, Death, Kidney diseases

## Abstract

**Introduction:** More studies are needed to examine whether race moderates the effect of baseline depressive symptoms on cause-specific mortality including deaths due to renal diseases in the United States.

**Objectives:** The present longitudinal study compared Blacks and Whites for the effect of baseline depressive symptoms on deaths due to renal diseases over a 25-year period in a nationally representative community sample.

**Patients and Methods:** Data came from the Americans’ Changing Lives (ACL) study, a nationally representative cohort that followed 3361 Black (n = 1156) or White (n = 2205) adults 25 and older for up to 25 years from 1986 to 2011. Month, year and cause of death were extracted from death certificates or national death index reports and coded based on ICD-9 or ICD-10 codes, depending on the year of death. We used Cox proportional hazards models for data analysis. Time to death due to renal diseases over a 25-year period was the outcome, baseline depressive symptoms (11-item Center for Epidemiological Studies-Depression [CES-D]) was the predictor, demographic characteristics, socio-economic status and chronic medical conditions (CMC) (hypertension, diabetes, chronic lung disease, heart disease, stroke, cancer, and arthritis) at baseline were controls, and race was the focal moderator.

**Results:** In the pooled sample, race and baseline depressive symptoms showed a significant interaction, suggesting a stronger effect of baseline depressive symptoms on deaths due to renal diseases for Whites compared to Blacks. In race-specific models, high depressive symptoms at baseline increased risk of death due to renal diseases among Whites but not Blacks.

**Conclusion:** The Black-White difference in the predictive role of baseline depressive symptoms on deaths due to renal diseases over a 25-year period found here provides support for the Black-White health paradox.

**Implication for health policy/practice/research/medical education:**

Findings of the current study have implications for the Black-White health paradox, which can be defined as lower prevalence of depression despite a higher burden of chronic medical conditions and other social and economic adversities among Blacks compared to Whites in the United States. Based on this paradox, a weaker association is expected between depression and medical disease among Blacks than Whites. However, no previous study has specifically tested if baseline depressive symptoms differently predict deaths due to renal diseases among Blacks and Whites.

## Introduction

Blacks have higher rate of chronic medical conditions (CMC) (1,2) such as hypertension (3), diabetes (4), and obesity (5) than Whites in the United States. As all these CMC are risk factors for subsequent chronic kidney disease (6), Blacks are also at higher risk of chronic kidney disease (5). Paradoxically, despite having a relatively high number of CMC, including chronic kidney disease, U.S. Blacks have lower rate of depression compared to U.S. Whites (7-12), a phenomenon known and studied in the United States as the Black-White health paradox. A growing body of evidence has documented supporting evidence of Black-White differences in the magnitude of the association between depression or depressive symptoms and CMC (13-25). To give one recent example, among Whites but not Blacks, high depressive symptoms at baseline increased the risk of CMC over time (12). With regard to renal outcomes more specifically, some studies have documented better survival and lower psychological distress among Blacks with chronic kidney disease when compared to Whites, despite their higher number of medical comorbidities (9), a pattern which aligns with the Black-White health paradox (10-12). However, very little is known about potential Black-White differences in the effect of baseline depressive symptoms on subsequent risk of renal disease or mortality from renal causes (20,26,27).

Although most of the literature on the link between depression and renal disease has conceptualized renal disease as the independent variable and depression as the dependent variable (28-30), the link between depression and CMC is bidirectional (12), suggesting that kidney disease should also be considered also as a potential outcome of depression (31-33). Although very limited, there is some evidence suggesting that baseline depression increases the risk of subsequent renal disease or progression of kidney disease to end stage renal disease (31-33). In addition, though it is clear that death can directly result from the progression of kidney disease and development of uremia (34,35), relatively few studies consider death due to renal disease as an outcome, at least partially because death due to renal diseases is not one of the leading causes of death (36).

More studies examining whether race moderates the effect of baseline depressive symptoms on deaths due to renal diseases in United States are needed, for at least two reasons. First and foremost, most studies on moderating effect of race on the link between depression and CMC have used general measures of CMC that simply count the number of conditions of all kinds (12,16,17), so we know relatively little specifically about renal disease and mortality from renal causes as outcomes (20). Second, most prior research on the differential link between depression and medical conditions has used a cross-sectional design, and those studies with prospective design have used a short term follow up period (13-19,21-25). This could be a limitation because the development of renal disease and its associated mortality may take a long time, so studies with long term follow up of the general population are needed (12,26,27).

## Objectives

To build on the existing literature on the race differences in the effects of depression on subsequent renal disease outcomes (12,29,31-33), we examined Black-White differences in the effects of baseline depressive symptoms on deaths due to renal diseases over a 25 year follow up in United States.

## Patients and Methods

### Setting

Data came from the Americans’ Changing Lives (ACL), a nationally-representative U.S. cohort study conducted from 1986 until 2011. Detailed information on the study design is available elsewhere (37,38).

### Sampling and participants

The ACL enrolled a stratified multistage probability sample of adults ages 25 or above who lived in the continental U.S. in 1986. The study included 3617 non-institutionalized respondents (representing 70% of sampled households and 68% of sample individuals at baseline) with an oversampling of those age 60 and older, and African Americans. Further interviews were conducted in 1989, 1994, 2001-2002 and 2011, but information from those interviews was not relevant for these analyses.

### Measures

Information on socio-demographic characteristics, depressive symptoms and CMC was measured at the baseline face to face interview in 1986.

### Socio-demographic characteristics

Demographic indicators included gender (a dichotomous variable with male as the referent category) and age (a continuous variable as number of years since birth), while socioeconomic status was measured with an indicator of education (less than 12 years of education, and 12 years or more). Race was the moderator, defined as non-Hispanic Black or non-Hispanic White based on a coding of self-reported items asking about Hispanic ethnicity, nativity, and racial category.

### Depressive symptoms

Baseline depressive symptoms were measured using the 11 item- Center for Epidemiological Studies-Depression scale (CES-D) (39). Items measured the extent to which respondents felt depressed, happy, lonely, sad, that everything was an effort, that their sleep was restless, that people were unfriendly, that they did not feel like eating, that people dislike them, that they could not get going, and that they enjoyed life. Positively worded items were reverse-coded. This abbreviated CES-D has shown acceptable reliability and a similar factor structure compared to the original version (40-42). Item responses were 1 (“hardly ever”) to 3 (“most of the time”). The mean score was computed across all 11 items, resulting in a continuous measure of depressive symptoms for baseline, with a potential range from 11 to 33. Higher scores indicated greater severity of depressive symptoms.

### Number of chronic medical conditions

Number of CMC at baseline was measured based on self-reported presence of seven CMC. All participants were asked whether a health care provider had ever told them they had each of seven focal conditions including hypertension, diabetes, heart disease, chronic lung disease, stroke, cancer, and arthritis. A sum score was calculated, potentially ranging from 0 to 7. A detailed description of the measurement of CMC is provided elsewhere (38). We controlled for CMC because some (i.e., hypertension and diabetes) cause renal disease and related death. As we were interested in the effect of depressive symptoms on renal disease death, we wanted to isolate this association, net of the association between depressive symptoms and CMC.

### Mortality due to renal diseases

The main outcome variables were mortality from renal diseases and time of death. Information on all deaths from mid-1986 through 2011 was obtained through the National Death Index (NDI), death certificates, and also from informants. In most cases, time and cause of death were verified with death certificates. The handful of cases where death could not be verified with death certificates were reviewed carefully, and actual death was certain in all cases. Only in these cases, was the date of death ascertained from the informants or the NDI report, rather than the death certificate. Cause of death was coded as unknown if death certificate or NDI report were unavailable.

We used the ICD 9 and ICD 10 codes (43), whichever was current at the time the death was recorded, to determine death due to renal diseases (kidney-urinary). For ICD -9 codes, we used codes 650 (acute glomerulonephritis and nephrotic syndrome), 660 (chronic glomerulonephritis, nephritis, and nephropathy, not specified as acute or chronic, and renal sclerosis, unspecified), 670 (renal failure, disorders resulting from impaired renal function, and small kidney of unknown causes), 680 (infections of kidney), and 690 (hyperplasia of prostate). For ICD-10 codes, we used the categorization of 113 selected causes of death provided by World Health Organization (WHO), for which codes 97 (nephritis, nephrotic syndrome, and nephrosis), 98 (acute and rapidly progressive nephritic and nephrotic syndrome), 99 (chronic glomerulonephritis, nephritis, and nephropathy not specified as acute or chronic, and renal sclerosis unspecified), 100 (renal failure), 101 (other disorders of kidney), 102 (infections of kidney), 103 (hyperplasia of prostate), and 104 (inflammatory diseases of female pelvic organ) were used. Respondents who died due to other causes were censored at the time of death. Time of death was registered as number of months from time of enrollment in the study to time of death, based on the month of death and the month of the baseline interview.

### Statistical analysis

Univariate, bivariate, and multivariable analyses were performed using Stata 13.0 (*Stata Corp*oration, College Station, TX, USA). A value of *P* < 0.05 was considered to be statistically significant. For multivariable analysis, Cox proportional hazards models were used to determine factors associated with time to death due to renal diseases over the 25 year follow up. Sample weights were applied in all analyses. Stratification and clustering in the estimation of standard errors was accounted for using Taylor series linearization. Sample sizes reflect the un-weighted sample distributions.

Cox proportional hazard models require information on an outcome (death due to renal diseases) and the time that outcome occurred since baseline (time to death due to renal diseases). Renal death was coded zero if the respondent did not die, or died from any other causes. Time to the renal death event, or to censoring, was defined as the number of months from baseline to death, loss to follow up, or the end of the year 2011. Model 1 included race, age, and gender. Education was added in Model 2. Model 3 added the count of CMC. In Model 4, we added the main effect of CES-D. Model 5 included an interaction term between race and depressive symptoms. Model 6 and Model 7 were race-specific models for Whites and Blacks, respectively. In these models, age, gender, education, CMC, and depressive symptoms were covariates. Hazard ratios with 95% CI are reported.

## Results

Table 1 shows descriptive statistics for the overall sample, and then separately for Whites and Blacks. Blacks were younger, had more CMC, and had higher depressive symptoms compared to Whites. Compared to Whites, Blacks had also lower frequency of high school graduation (All these differences by race were significant at *P* < 0.05). Death due to renal disease was also more common among Blacks than Whites.

**Table 1:** Descriptive statistics of socio-demographic characteristics, chronic medical conditions, and depressive symptoms overall and among Blacks and Whites at baseline

	**All**		**Whites**		**Blacks**	
	Mean (SE)	95% CI	Mean (SE)	95 % CI	Mean (SE)	95 % CI
Age	47.79 (0.53)	46.72-48.86	47.98 (0.60)	46.77-49.19	46.37 (0.71)	44.93-47.81
CMC	0.80 (0.03)	0.74-0.85	0.78 (0.03)	0.71-0.84	0.92 (0.05)	0.82-1.02
CES-D (z score)	-0.03 (0.02)	-0.08-0.02	-0.07 (0.03)	-0.13--0.02	0.28 (0.05)	0.18-0.38
	% (SE)	95% CI	% (SE)	95% CI	% (SE)	95% CI
Gender						
Male	47.26 (0.01)	44.86-49.68	47.82 (0.01)	45.12-50.52	43.18 (0.02)	38.79-47.69
Female	52.74 (0.01)	50.32-55.14	52.18 (0.01)	49.48-54.88	56.82 (0.02)	52.31-61.21
Education > 12 years						
No	23.93 (0.01)	21.37-26.70	21.71 (0.01)	18.87-24.85	40.25 (0.03)	34.55-46.24
Yes	76.07 (0.01)	73.30-78.63	78.29 (0.01)	75.15-81.13	59.75 (0.03)	53.76-65.45
Renal death						
No	99.48 (0.00)	98.97-99.74	99.56 (0.00)	98.94-99.82	98.85 (0.00)	97.91-99.37
Yes	0.52 (0.00)	0.26-1.03	0.44 (0.00)	0.18-1.06	1.15 (0.00)	0.63-02.09

Abbreviations: CES-D, Center for Epidemiologic Studies Depression Scale; CMC, chronic medical conditions.

Table 2 shows results from the Cox proportional hazard regression models in the pooled sample (Models 1-5) or by race (Models 6-7). According to the results of Model 1, Blacks were at higher risk of death due to renal disease, even adjusting for the effects of age and gender. The hazard for Black respondents remained significant in Model 2, after adjusting for education and also in Model 3, after adjusting for the count of CMC. The results of Model 4 show that more depressive symptoms were linked to a significantly higher hazard of death due to renal disease; while the hazard for Black respondents remained higher than that for Whites after adjusting for depressive symptoms, the difference was now only marginally significant. In Model 5 we added an interaction term between race and depressive symptoms to test whether the impact of depression was greater or less for Blacks as compared to Whites. The results for Model 5 show that there was a significant interaction, and that for Blacks, the depression-renal mortality link was significantly weaker. As a further test for differences by race, we ran stratified models; Model 6 was the race specific model for Whites, and results showed that greater depressive symptoms were significantly associated with a higher hazard of death due to renal disease. Results for Model 7, the race-specific model for Blacks, revealed no association between depressive symptoms at baseline and subsequent death due to renal disease.

**Table 2:** Results of Cox regressions predicting death due to renal disease among White and Black respondents from 1986 to 2011 as a function of depressive symptoms in 1986 and other factors

	**All**					**Whites**	**Blacks**
	**Model 1 (All)** **Demographics**	**Model 2 (All)** **M1 + Education**	**Model 3 (All)** **M2 + CMCs**	**Model 4 (All)** **M3 + CES-D**	**Model 5 (All)** M4 + CES-D * Black	**Model 6 (Whites) M4**	**Model 7 (Blacks) M4**
	**HR (95% CI)**	**HR (95% CI)**	**HR (95% CI)**	**HR (95% CI)**	**HR (95% CI)**	**HR (95% CI)**	**HR (95% CI)**
Black	3.09^a^(1.02-9.37)	3.08^a^(1.18-8.07)	2.8^a^(1.08-7.28)	2.61^d^(0.97-7.00)	4.11^b^(1.59-10.63)	-	-
Age	1.05^c^(1.03-1.07)	1.05^C^(1.03-1.07)	1.03(0.99-1.06)	1.03^a^(1.01-1.07)	1.03^a^(1.01-1.07)	1.03^d^(1.00-1.07)	1.04(0.98-1.11)
Female	0.67 (0.17-2.7)	0.67(0.16-2.83)	0.58(0.13-2.53)	0.52(0.11-2.38)	0.52(0.11-2.37)	0.31(0.05-1.86)	2.49(0.66-9.44)
Education (>12 y)		0.99(0.29-3.4)	1.28(0.31-5.2)	1.68(0.34-8.28)	1.71(0.36-8.07)	1.93(0.20-18.45)	1.39(0.52-3.70)
CMC			1.93^a^(1.14-3.25)	1.74^a^(1.04-2.91)	1.72^a^(1.04-2.85)	1.81^a^(1.01-3.23)	1.38(0.70-2.73)
CES-D				1.66^b^(1.19-2.32)	1.96^C^(1.46-2.63)	2.02^C^(1.45-2.82)	0.94(0.60-1.50)
CES-D × Black					0.51^b^(0.36-0.74)	-	-

Abbreviations: CES-D, Center for Epidemiologic Studies Depression Scale; CMC, chronic medical conditions. ^a^*P* < 0.05; ^b^*P* < 0.01; ^c^ *P* < 0.001; ^d^*P* < 0.1.

## Discussion

According to our findings, baseline depressive symptoms predict renal disease mortality among Whites but not Blacks in a nationally-representative sample of U.S. adults. This is in line with the literature that supports the Black-White health paradox, a hypothesis suggesting that Blacks and Whites differ in their cross-sectional and longitudinal associations between depression and CMC (1,2,7,8,10,12-16,19,25). For example, higher depressive symptoms at baseline were predictive of incident CMC among Whites but not Blacks in a recent study (12). In another study, baseline depressive symptoms predicted subsequent cardiovascular mortality among Whites but not Blacks, after adjusting for covariates (44). Thus, the racial heterogeneity we find here in the magnitude of the longitudinal association between depressive symptoms and renal disease death in the United States is in line with the Black-White health paradox (10,12).

While supportive evidence is accumulating (45,46), it is important to note that the body of evidence for the link between depression and CMC shows some mixed (16-18,47), and even some unexpected findings (15,16,48) that contradict the Black-White health paradox (15,48). For instance, Hankerson and colleagues reported a stronger link between major depression and hypertension and obesity among Blacks compared to Whites (48) and Assari found a cross-sectional positive association between major depressive disorder and hypertension among Blacks but not Whites (25). Watkins and colleague (17) also showed that depression was associated with at least one chronic medical condition among Blacks but not Whites. More pertinent for our own analysis, some studies have failed to show any moderating effect of race on the association between depression and CMC including kidney disease, hypertension, and diabetes (16,20). For example, in a cross-sectional study, Assari did not find a moderating effect of race on the depression – kidney disease link (20). That study however, focused on self-reported kidney disease but not mortality due to kidney disease, whereas the latter is the focus of the present paper.

Our findings lend support to other recent work; a study of patients with chronic kidney disease showed that depression predicted progression toward end stage renal disease (32). Interestingly, another study showed that depression predicted subsequent hypertension, heart disease, arthritis, asthma, back pain, chronic respiratory condition, and migraines, but not kidney disease (46). However, that study examined incidence of kidney disease, whereas other studies have shown that depression may worsen the course of existing CMC and influence the risk of death from that disease (49). Our finding for a greater risk of death due to renal causes among Whites with higher levels of depressive symptoms, compared to that for Whites with lower levels of depressive symptoms, extends the previous literature by showing an increased risk of death from a specific and less-studied cause (49). Our findings thus suggest that the role of depressive symptoms on development or progression or death due to renal disease may depend on race, with larger effects among Whites than Blacks.

Our results showed a larger effect of depression on renal disease mortality among Whites than Blacks, clearly countering the “accelerated risk” hypothesis that suggests the opposite: a greater influence of depressive symptoms on subsequent CMC such as heart disease among Blacks, as compared with Whites (13,50). It is also difficult to explain our findings using the literature suggesting that depression is more chronic and disabling for Blacks compared to Whites (8,17). If depression is more disabling among Blacks than Whites, we would expect more negative consequences of depression for Blacks (12) in terms of their renal mortality outcomes. However, we observe the reverse finding. To explain Black-White differences in the association between depression and CMC, Jackson has hypothesized that Blacks’ engagement in coping via negative health behaviors (e.g., smoking or drinking) may weaken the association between CMC and psychopathology including depression (21-23,51-53). The same hypothesis may help to explain why depressive symptoms have a weaker effect on mortality due to renal disease among Blacks than Whites. Although Jackson’s hypothesis is not limited to any specific medical condition, coping behaviors such as smoking and drinking have implications for chronic kidney diseases and their associated mortality (54,55).

Even though the Black-White health paradox and the findings of the present analysis suggest that depression is less consequential for Blacks facing serious chronic conditions than for their White counterparts, this does not mean that we can ignore crucial racial disparities in the diagnosis and treatment of depression. Given wide range of factors such as low access and trust, high stigma, and endorsement of more negative beliefs regarding pharmaceutical treatment of depression, Blacks are less likely to receive diagnosis and treatment among Blacks than Whites (8,17,56). These gaps highlight the importance of racially and culturally tailored interventions that may promote depression detection and treatment in the United States (57-59). Blacks are also more likely to receive lower quality treatment for depression in the primary care setting (60-63). Comorbid medical conditions add to the complexity of diagnosis of depression among Blacks (1,2), especially because health care providers believe that depression is more somatic among Blacks (64). These disparities all highlight the need for further research on race differences in the links between depression and CMC.

## Limitations of the study

This study makes a unique contribution to the literature by extending our current understanding of the Black-White health paradox (12) to the effect of depression on renal causes of death at the population level (31-33). However, the results should be interpreted with consideration of the limitations. First, this study did not have any measure of baseline kidney disease, and only used a total number of medical conditions at baseline. In addition, measurement of baseline medical conditions was based on self-reported data, which is subjected to recall bias (65). Furthermore, our list of medical conditions was not very comprehensive and did not include kidney disease. In addition, the study included symptoms of depression, not clinical depression. Future studies should assess whether clinically-diagnosed depression differently predicts decline in kidney function and trajectory of patients with end stage renal disease in particular among Blacks and Whites. Despite these limitations, the study had major strengths, including a long term follow up, a nationally representative U.S. sample and a large sample of Blacks, enhancing statistical power in a study of a relatively rare outcome.

## Conclusion

To conclude, we found Black-White differences in the predictive role of baseline depressive symptoms on deaths due to renal diseases over a 25-year period. This finding extends the growing evidence for the Black-White health paradox to a new and important outcome measure. Our findings are particularly important because of the Black-White disparity in incidence of burden of chronic kidney disease and associated mortality in the United States (66).

## Authors’ contribution

SA designed this particular research question. SB conducted the mother research and collected the data. SA analyzed the data. SA and SB collaboratively prepared the manuscript. Both authors read, revised, and approved the final manuscript.

## Conflicts of interest

Authors declare that they have no conflicts of interest.

## Ethical considerations

All procedures followed were in accordance with the ethical standards of the responsible committee on human experimentation (institutional and national) with the Helsinki Declaration of 1975, as revised in 2000. Informed consent was obtained from all participants included in the study. Ethical issues (including plagiarism, misconduct, data fabrication, falsification, double publication or submission, redundancy) have been completely observed by the authors.

## Funding/ Support

The Americans’ Changing Lives (ACL) study was supported by Grant # AG018418 from the National Institute on Aging (DHHS/NIH), and per the NIH Public Access Policy requires that peer-reviewed research publications generated with NIH support are made available to the public through PubMed Central. NIH is not responsible for the data collection or analyses represented in this article. The ACL study was conducted by the Institute of Social Research, University of Michigan.
